# Association of Climate Variables with *Plasmodium vivax* and *Plasmodium falciparum* Malaria Cases in Mandoto, Madagascar: A Statistical Modeling Study

**DOI:** 10.4269/ajtmh.25-0329

**Published:** 2026-02-10

**Authors:** Eliharintsoa Rajaonarimirana, Masiarivony Ravaoarimanga, Sophie Lockwood, Estee Cramer, Malalatiana Rajesy, Chris Drakeley, Rindra Vatosoa Randremanana, Michael White

**Affiliations:** ^1^ Epidemiology and Clinical Research Unit, Institut Pasteur de Madagascar, Antananarivo, Madagascar;; ^2^ Infectious Disease Epidemiology and Analytics G5 Unit, Institut Pasteur, Université Paris Cité, INSERM, Paris, France;; ^3^ Collège Doctoral, Sorbonne Université, Paris, France;; ^4^ Department of Ecology and Evolution, University of Chicago, Chicago, Illinois;; ^5^ Mandoto Public Health District Service, Antananarivo, Madagascar;; ^6^ Department of Infection Biology, London School of Hygiene & Tropical Medicine, London, United Kingdom

## Abstract

The Mandoto District in the central highlands of Madagascar experiences year-round transmission of *Plasmodium vivax* (*P. vivax*) and *Plasmodium falciparum* (*P. falciparum*). Monthly malaria case data from 27 health centers across Mandoto between 2019 and 2024 were analyzed alongside meteorological data to understand transmission dynamics and forecast potential influences of climate change using descriptive, cross-correlation, and seasonal autoregressive integrated moving average forecast models. Over a period of 6 years, 276,318 rapid diagnostic tests (RDTs) were performed, yielding a 39.6% positivity rate, totaling 109,428 malaria cases. After 2021, when multispecies RDTs became available, 71.5% of cases were attributed to *P. falciparum*, and 28.5% were attributed to *P. vivax*. Both species were co-endemic across all health centers, with the western region experiencing a higher transmission risk. Malaria cases peaked in January, with a second peak from April to June after the rainy season, and declined between July and September. Precipitation and temperature effectively revealed the seasonality of malaria dynamics, thereby improving model accuracy. *Plasmodium falciparum* exhibited stronger associations with precipitation and temperature variability. The present study highlights that combining time-series modeling with precipitation and temperature data can help predict malaria cases and support timely planning and resource allocation.

## INTRODUCTION

In Madagascar, an island nation off the southeast coast of Africa, malaria is a significant public health concern. It was the fourth leading cause of morbidity at basic healthcare facilities and the fourth leading cause of death in hospitals.^1^ There has been notable variation in malaria transmission levels in Madagascar over the past decade, with substantial increases since 2019. These increases have been linked to multiple factors, including climate effects, such as cyclones and heavy rainfall, which limit access to healthcare, and changes in surveillance practices that may have improved case detection.^2^^,^^3^ In 2022, Madagascar experienced an estimated 3.5 million malaria cases, resulting in 9,111 deaths.^4^ Four malaria species are present in Madagascar, with *Plasmodium falciparum* (*P. falciparum*) being the most prevalent, followed by *Plasmodium vivax* (*P. vivax*).^5^^–^^7^ In its dormant hypnozoite form, *P. vivax* can lead to relapse of the disease weeks, months, or even years after initial infection. Relapses account for 60–90% of recurrent infections and clinical cases of *P. vivax* malaria.^8^ Malaria transmission in Madagascar is driven by *Anopheles* mosquitoes, with multiple Anopheline species identified as putative vectors across the country. *Anopheles gambiae*, *Anopheles funestus* (*An. funestus*), and *Anopheles arabiensis* are the primary species that facilitate the spread of the disease.^9^^–^^11^ Malaria dynamics are highly sensitive to environmental conditions. Mosquito life history traits related to malaria transmission, including biting rate, development rate, survivorship, and vector competence, are dependent on temperature, with trait values maximized at intermediate temperatures.^12^^,^^13^ The biology of malaria parasites is also influenced by temperature, with parasite development within mosquito hosts being dependent on ambient temperature.^14^ Additionally, moderate rainfall can create and sustain breeding habitats for mosquitoes; however, excessive or intense rainfall may destroy larval habitats or reduce adult survival, thereby slowing transmission.^15^^,^^16^

In Madagascar, the dependence of malaria transmission on environmental and climatic factors varies geographically, driven by regional differences in precipitation, temperature, humidity, and altitude.^17^^–^^21^ In the Vakinankaratra Region, located in the central highlands of Madagascar, malaria is primarily transmitted by *An. funestus*, and *P. vivax* infection is common.^5^^,^^9^ Among Vakinankaratra’s seven districts, five are classified as highland malaria zones, whereas the remaining two, including the Mandoto District, are considered fringe zones, characterized by warmer climates and more favorable conditions for mosquitoes.^9^ Mandoto experiences ongoing malaria transmission throughout the year, with *An. funestus* identified as a key vector for malaria.^22^^,^^23^ Furthermore, the area is co-endemic for *P. vivax* and *P. falciparum*, and transmission of both species is shaped by the pronounced wet and dry seasonal cycle in Madagascar.^24^

Control programs and the authors of previous studies in Madagascar have typically concentrated on *P. falciparum* because of its higher prevalence and more severe disease outcomes, or they have addressed malaria in general without distinguishing between species.^25^^–^^28^ However, in regions with multiple parasite species, it is beneficial for control and elimination programs to target all species.^29^ Heterogeneity in transmission dynamics across Madagascar underscores the importance of investigating the epidemiology and dynamics of each *Plasmodium* species within districts, an analysis enabled by data from individual health center reports.

The aim for the present study is to enhance the current understanding of malaria epidemiology in Mandoto by conducting a time-series analysis of overall malaria cases spanning the years 2019–2024, with a focus on species-specific data for 2021–2024. By examining both *P. vivax* and *P. falciparum*, which are highly prevalent in the district, the aim is to assess the transmission dynamics of each species and evaluate the impact of climate variables, such as temperature and precipitation, on malaria transmission patterns. This is the first study in which time-series data are analyzed by species using routine health center testing in Madagascar, offering valuable insights for more targeted malaria control.

## MATERIALS AND METHODS

### Study design and area.

**Figure 1.**
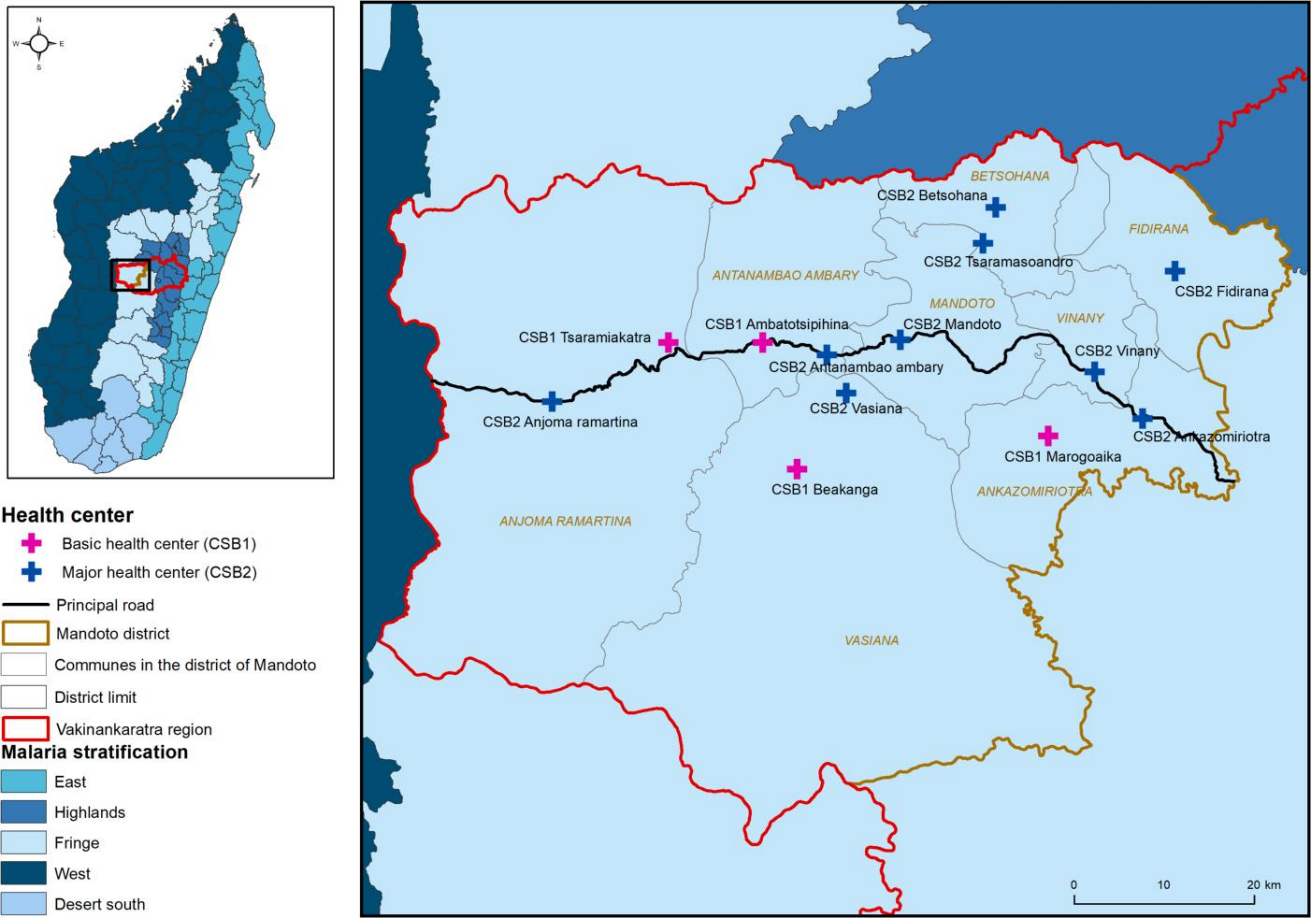
Mapping of the eight communes in the Mandoto District of Madagascar with major (blue, CSB2) and basic (pink, CSB1) health center locations labeled. Centre de Santé de base (CSB) refers to a primary health center. A map of Madagascar (inset) reveals the Vakinankaratra Region outlined in red and includes malaria stratification (Fringe and Highlands) by color.

The present retrospective study was conducted in the Mandoto District, located in the central highlands of Madagascar, in the western part of the Vakinankaratra Region. This district is situated between –19.396°S and –20.108°S latitude and 45.716°E and 46.719°E longitude. Mandoto covers an area of 4,500 km^2^ and has the lowest population density of all districts in the region, with 43 inhabitants per square kilometer according to the most recent national census conducted in 2018.^30^ The primary activities in the area include rice cultivation and livestock farming. The district is also known for its mineral extraction caves, where men work during the off-season to supplement their income, particularly in the western part of the district. Mandoto is divided into eight communes, and its residents receive care at 27 health centers, comprising 15 public and 12 private health facilities, which specialize in services such as maternity (Figure 1 and Supplemental Figure 1). The district is classified as an unstable malaria transmission area. Approximately 50% of the population in the region is Duffy-positive, which makes them susceptible to *P. vivax* infections.^31^

### Data collection.

Malaria data were collected from the Mandoto Public Health Service District through paper-based monthly reports from January 2019 to December 2023 across all health centers within the district. Data from 2024 were only available in aggregated form at the district level. These reports, submitted by each health center to the health district, captured rapid diagnostic test (RDT) results for patients with suspected malaria infections who were seeking medical care.^24^ A confirmed malaria case is defined as a person who has a positive malaria diagnosis made via an RDT.

Between 2019 and 2020, the monthly reported malaria cases were not recorded by species and were grouped into four age classes: less than 1 year, between 1 and 5 years, between 6 and 13 years, and greater than 14 years. Starting in January 2021, the health centers began to differentiate between infections caused by *P. falciparum* and those caused by non-*falciparum* malaria using RDTs, and an additional age class (i.e., older than 25 years) was added. The *P. falciparum* or mixed *P. falciparum* and other species malaria cases (RDT 111) were grouped with *P. falciparum* cases (RDT 101), whereas non-*falciparum* cases (RDT 110) were assumed to be *P. vivax* cases on the basis of previous work, which revealed that non-*falciparum* cases in the study area are predominantly due to *P. vivax.*^25^ Population data were estimated using the latest census from the National Institute of Statistics, conducted in 2018, with a fixed 3.01% national annual growth rate applied, and grouped by commune.^30^

Meteorological data for the study period were obtained from the ERA5-Land dataset (https://cds.climate.copernicus.eu/datasets/reanalysis-era5-land?tab=overview), which provides hourly temperature and total precipitation data at a spatial resolution of 9 km. Hourly temperatures were used to calculate daily minimum and maximum temperatures. Monthly minimum and maximum temperatures were calculated by averaging the corresponding daily values, whereas the total monthly precipitation was aggregated from the daily sums for analysis. To ensure reliability, ERA5 estimates were compared with available station measurements (2019–2021) from the Service of the Data Bank and Hydrometeorological Archives of Madagascar before use, with the figures exhibiting close agreement.

## STATISTICAL ANALYSES

The Annual Parasite Incidence (API) was calculated by dividing the total number of cases per commune per year by the estimated population of each commune, then multiplying by 1,000. The API was categorized according to the risk stratification in Madagascar’s National Strategy Plan: high-risk (incidence >100 cases per 1,000), medium-risk (incidence between 1 and 100 per 1,000), and low-risk (incidence less than 1 per 1,000).^32^ To allow for a proportional comparison across species, the data were normalized by dividing the number of cases per species in each month by the total number of cases for the year.

A descriptive analysis of malaria incidence, both by species and overall, was conducted by summarizing data at the Mandoto District and commune levels and plotting incidence over time. The seasonal trend decomposition (STL) based on locally weighted regression was used to compare the seasonality of *P. vivax* and *P. falciparum* using the monthly proportion of malaria cases, normalized by the total number of malaria cases in the year.^33^ In the STL method, the observed time series is separated into three components: seasonal patterns, trends, and residuals. This allows for the examination of monthly variations in case proportions and a better understanding of seasonal dynamics. The stationarity of the time series was assessed by analyzing autocorrelation function (ACF) plots and partial ACF plots. Time series decomposition and analysis were conducted using the stats package in R (R Foundation, Vienna, Austria).

Separate seasonal autoregressive integrated moving average (SARIMA) models were developed for log-transformed malaria incidence for each species (*P. vivax* and *P. falciparum*), as well as for the overall dataset to model the temporal dynamics of malaria incidence, assess the relationship between district-level malaria incidence and climate variables, and predict malaria trends.^34^ Data from 2019 through 2023 were used to develop the SARIMA model, and data from 2024 were used for model validation. An initial univariate SARIMA model was fitted to the malaria time series to account for trend and seasonality. The best model was selected using the auto.arima function of the forecast R package on the basis of the minimum Akaike information criterion (AIC) value and diagnostic checks.^35^^–^^39^ To assess the influence of climate variables on malaria incidence, cross-correlation analysis (CCA) was first performed on the raw time series to identify potentially significant lags. To determine if these correlations were driven by shared seasonality or autocorrelation, prewhitening was then applied to all the series.^37^^,^^40^ If prewhitening confirmed significant correlations, the identified lags were incorporated into the selected univariate SARIMA models as an external regressor. If no significant correlations remained after prewhitening, this indicated that the apparent relationships were due to shared seasonal patterns rather than direct effects. In that case, seasonal autoregressive integrated moving average with exogenous regressors (SARIMAX) models were fitted using the auto.arima function on the log-transformed malaria incidence series, testing all possible combinations of lags for precipitation, minimum temperature, and maximum temperature suggested by the initial CCA as external regressors. Models were fitted using data from 2019–2023 and validated against data from 2024, with the final model selected on the basis of the minimum mean absolute error (MAE). In May 2024, rapid diagnostic testing was disrupted: only 33% of febrile individuals were tested, and among those tested, 62% were positive. Because overall RDT coverage among febrile patients in 2024 was 94% on average, the number of positive RDT results in May 2024 was adjusted to reflect 62% positivity if testing coverage had included 94% of the population, applying this adjustment before model validation.

The SARIMA model is defined according to parameters that capture nonseasonal and seasonal influences and is expressed as (p, d, q)(P, D, Q)*_s_*, where p is the order of the autoregressive (AR) polynomial, q is the order of the moving average (MA) polynomial, d is the degree of differencing, P is the order of the seasonal AR polynomial, Q is the order of the seasonal MA polynomial, D is the degree of seasonal differencing, and s is the seasonal period.^41^^–^^43^ The adequacy of the final models was evaluated by analyzing residuals using ACF plots, the Ljung-Box Q test, and out-of-sample metrics. Large *P*-values from the Ljung-Box test indicate a well-fitted model.^44^ To assess the robustness of the district-level models, a commune-level sensitivity analysis was performed by refitting the best identified SARIMA model separately for each commune. The direction of the coefficients and scaled MAE were compared across communes to evaluate whether model performance and associations remained consistent. All analyses were conducted using R version 4.4.2.

## RESULTS

### Epidemiological profile of malaria in the Mandoto District.

During the 72-month study period (January 2019 to December 2024), a total of 276,318 RDTs were performed at 27 healthcare facilities across the eight communes in the district, with a positivity rate of 39.6% (109,428 cases; Table 1). A total of 83,632 malaria cases were reported between January 2021 and December 2024. Among these, 14 cases lacked species identification and were excluded from the species analysis. Excluding these, 83,618 cases were classified by species, with 59,749 (71.5%) attributed to *P. falciparum* and 23,869 (28.5%) attributed to *P. vivax* (Table 1). The number of annual malaria cases in the Mandoto District ranged from 6,230 in 2019 to 26,327 in 2024, with the highest number of cases reported in 2024. However, the highest API was observed in 2021, with 110 cases per 1,000 population, equivalent to 25,118 cases, compared with 106 cases per 1,000 population in 2024. *Plasmodium vivax* and *P. falciparum* are co-endemic in all communes within the district, as shown in Table 1. The number of reported *P. vivax* cases per month ranged from 1,027 to 3,189, whereas the number of *P. falciparum* cases ranged from 1,671 to 8,928.

**Table 1:** Annual breakdown of malaria cases by commune and species (*Plasmodium vivax* and *Plasmodium falciparum*) from 2019 to 2024 in the Mandoto District

Commune	2019 Cases (*Pv|Pf*)	2020 Cases (*Pv|Pf*)	2021 Cases (*Pv|Pf*)	2022 Cases (*Pv|Pf*)	2023 Cases (*Pv|Pf*)	2024 Cases (*Pv|Pf*)
Mandoto	927 (–|–)	2015 (3|15)*	2,368 (787|1,581)	1,525 (401|1,124)	1,267 (445|822)	–
Antanambao Ambary	393 (–|–)	2,439 (–|–)	4,087 (1,204|2,883)	2,778 (1,025|1,753)	3,949 (1,119|2,830)	–
Betsohana	398 (–|–)	1,517 (–|–)	1,731 (442|1,289)	607 (199|408)	768 (153|615)	–
Anjoma Ramartina	3,497 (–|–)	7,408 (–|–)	9,222 (2,118|7,104)	5,765 (2,069|3,696)	5,867 (1,874|3,993)	–
Vinany	193 (–|–)	871 (–|–)	1,335 (78|1,257)	195 (14|181)	274 (23|251)	–
Fidirana	61	971 (55|–)*	776 (545|231)	191 (116|75)	124 (17|107)	–
Vasiana	183 (–|–)	1,737 (–|–)	3,062 (387|2,675)	2,040 (568|1,472)	2,176 (474|1,702)	–
Ankazomiriotra	578 (–|–)	2,608 (–|–)	2,537 (488|2,035)^†^	695 (99|596)	633 (70|563)	–
Total	6,230 (–|–)	19,566 (58|15)	25,118 (6,049|19,055)	13,796 (4,491|9,305)	18,391 (5,108|13,283)	26,327 (8,221|18,106)

*Pf* = *Plasmodium falciparum*; *Pv* = *Plasmodium vivax.*

* Health centers reported rapid diagnostic test results with species identification in December 2020, recording 73 cases.

^†^ Health centers reported rapid diagnostic test results using the previous format (without species identification), recording 14 cases.

**Figure 2.**
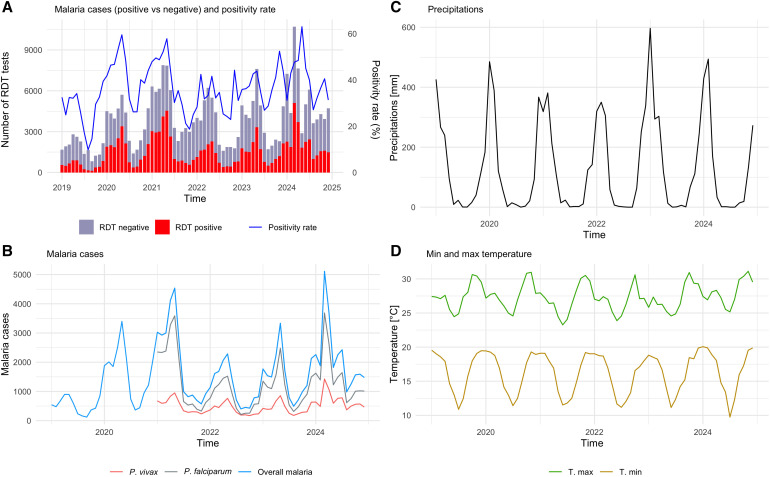
Temporal distribution of malaria cases reported from health facilities throughout the Mandoto District of Madagascar from January 2019 to December 2024 and meteorological data for the Mandoto Region from the ERA5 dataset. (**A**) Number of malaria rapid diagnostic tests stratified by positivity status, with positivity rate per month. (**B**) Temporal distribution of overall malaria cases and per species. (**C**) Total monthly precipitation in mm. (**D**) Monthly minimum and maximum temperatures in °C.

Regarding climate data derived from satellite observations, Mandoto experiences annual wet and dry seasons. The wet season, characterized by the warmest temperatures, occurs from November to April, and the dry season, with cooler temperatures, occurs from May to October. During the study period, temperatures in the Mandoto District ranged from 9.7°C to 31.1°C, and total monthly precipitation ranged from 0 mm to 596.5 mm. July is typically the coldest month, whereas November is the warmest (Figure 2). Spatially, a clear temperature gradient was observed across communes: the western part of the district is consistently warmer, whereas the eastern part is cooler. In Anjoma Ramartina, located furthest west, temperatures ranged from 11.6°C to 32.6°C, whereas in Ankazomiriotra, in the east, they ranged from 8.5°C to 29.9°C (Supplemental Figure 2).

The temporal distribution of overall malaria cases and cases per species is shown in Figure 2. The highest number of malaria cases occurred in May during the dry season, whereas the fewest were observed between July and September. Notably, there were two peaks in annual malaria cases, the first in January and the second in May (Supplemental Figure 3).

**Figure 3.**
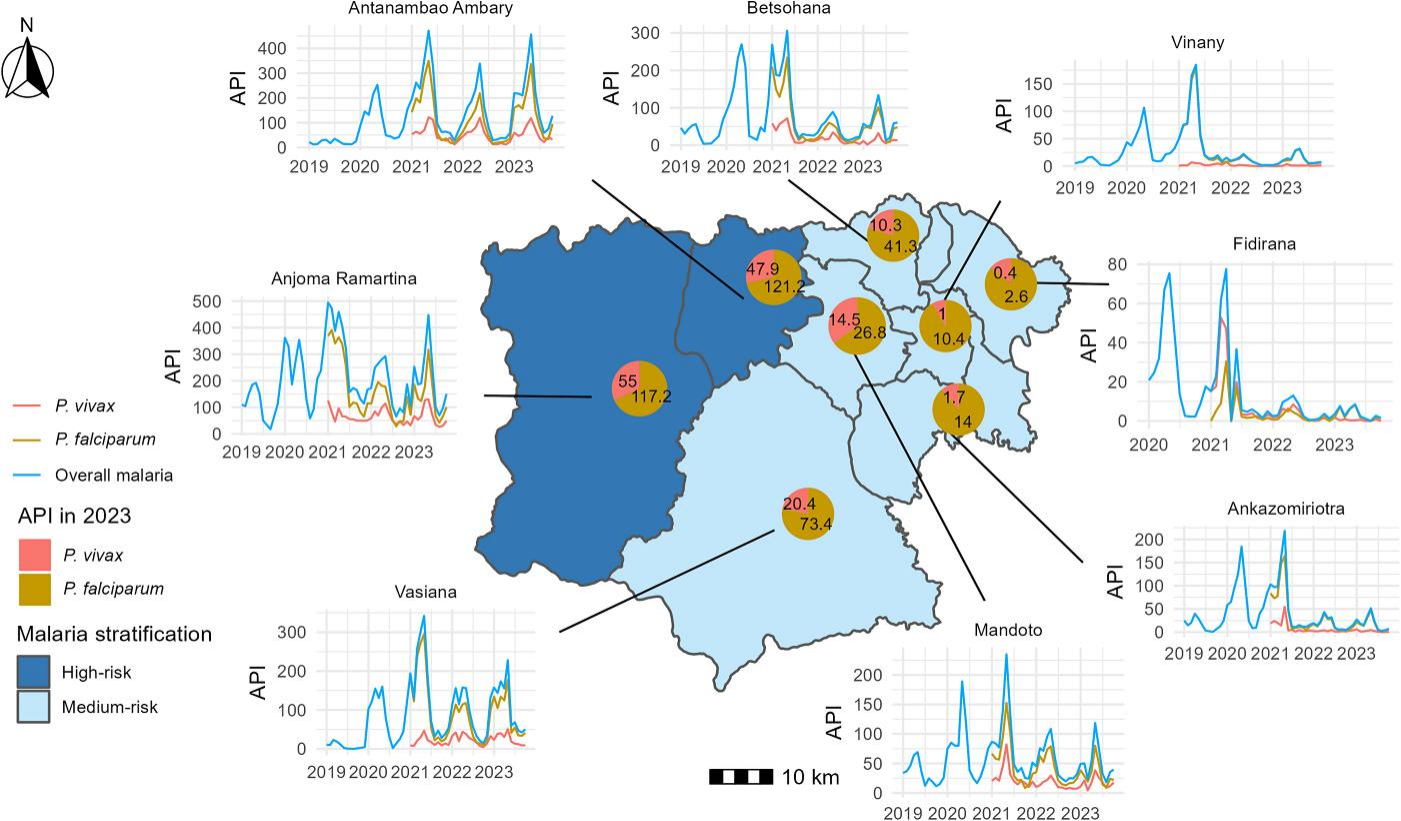
Mapping of malaria incidence by commune within the Mandoto District. Commune-level annual parasite incidence (the number of cases in a commune divided by that commune’s estimated population and scaled by 1,000) is displayed for each month between 2019 and 2023. Malaria stratification is represented in the map’s background color. Medium-risk malaria corresponds to an annual incidence of 1 to 100 per 1,000, and high-risk malaria corresponds to an annual incidence greater than 100 per 1,000. Data were provided by the Mandoto Public Health District Service on the basis of monthly reports from all health centers within the district from 2019 to 2023. Population data were estimated from the latest National Institute of Statistics census in 2018, using a fixed national annual growth rate of 3.01%.

Spatial variation in the malaria burden was observed in the Mandoto District. The westernmost commune, Anjoma Ramartina, had the highest malaria incidence, with the number of *P. vivax* cases ranging from 55 to 66 per 1,000 population and the number of *P. falciparum* cases ranging from 117 to 221 per 1,000 population. In contrast, the Fidirana had the lowest number of malaria cases, ranging from 2 to 25 per 1,000 population, and was the only commune where *P. vivax* was more prevalent than *P. falciparum* (Figure 3). Fewer cases were reported from private health centers because of a combination of factors, including their specialization in maternity care and recent establishment.

### Seasonality of *P. vivax* and *P. falciparum* proportion.

**Figure 4.**
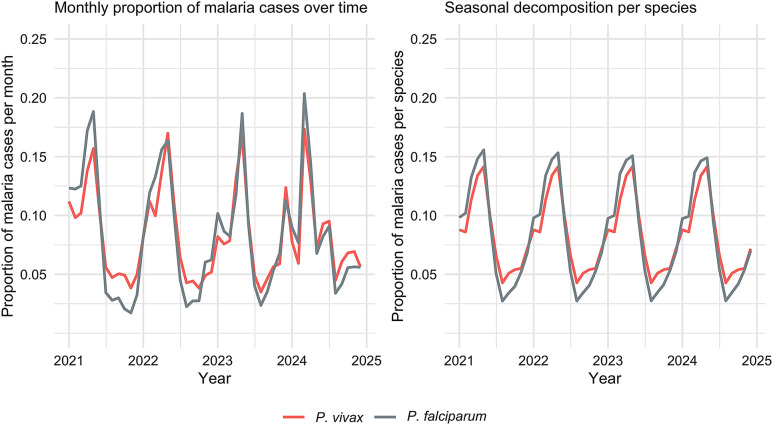
The figure on the left represents the distribution of the monthly proportion of malaria cases (*Plasmodium vivax* [*P. vivax*; red] and *Plasmodium falciparum* [*P. falciparum*; gray]). The monthly proportion is calculated by dividing the number of cases in each month by the total number of cases over the entire year. The figure on the right highlights the seasonality of *P. vivax* and *P. falciparum* proportion cases obtained through seasonal trend decomposition (STL) using locally weighted regression (STL).

The proportion of malaria cases caused by *P. vivax* or *P. falciparum* in a given month, normalized by the total annual malaria cases, along with the seasonality decomposition of the proportion, is presented in Figure 4. The time series reveals clear seasonal variation, with a repeating cycle every 12 months, corresponding to annual fluctuations. The autocorrelation coefficient at a 12-month lag in Supplemental Figure 4 has a high positive value, indicating strong seasonality. Both *P. vivax* and *P. falciparum* exhibit synchronized peaks at the beginning of the year and in May, with an additional modest increase in *P. vivax* cases in September. The average proportion of cases occurring in May was 14.2% for *P. vivax* and 15.2% for *P. falciparum*. The deviation of the proportion of *P. vivax* from the overall mean (8.4%) in May is 5.9%, whereas *P. falciparum* exhibits a deviation of 6.9% from its overall trend (8.4%). Although the peaks in May are almost identical, their behavior during the trough period in August and September reveals a notable difference. In August, the proportion of *P. vivax* cases declines less than that of *P. falciparum*, with *P. falciparum* cases decreasing by 5.6% from the overall trend in the proportion, whereas *P. vivax* cases decrease by 4.1%.

### Separate seasonal autoregressive integrated moving average models.

The most effective model for evaluating overall malaria incidence is SARIMA (0,1,0) (0,1,1)_12_ (baseline model), selected for its lowest AIC value (25.83) among the models. For *P. vivax*, the most effective model according to AIC was SARIMA (1,1,0) (0,1,0)_12,_ whereas for *P. falciparum*, the most effective model was SARIMA (0,1,0) (1,1,0)_12_ (Table 2).

**Table 2:** Comparison of seasonal autoregressive integrated moving average models of overall, *Plasmodium vivax*, and *Plasmodium falciparum* log-transformed malaria incidence with and without covariates, including coefficients and statistical significance

Model	ar1	sar1	sma1	Tp [1]	Tp [3]	Tp [4]	Tmax [0]	Tmax [1]	Tmax [2]	Tmax [3]	Tmin [3]
SARIMA models of overall malaria											
Baseline (0,1,0) (0,1,1)_12_	–	–	0.602 (0.240)*	–	–	–	–	–	–	–	–
(0,1,0) + Tp [1,3,4] + Tmax[0,2]	–	–	–	0.001 (0.0005)	0.001 (0.0004)*	0.002 (0.0005)*	−0.013 (0.0351)	–	0.111 (0.0339)*	–	–
SARIMA models of *P. vivax*											
Baseline (1,1,0) (0,1,0)_12_	−0.542 (0.2229)*	–	–	–	–	–		–	–	–	–
(0,1,0) + Tp [1,3] + Tmax [2]	–	–	–	0 (0.0005)	0.002 (0.0005)*	–		–	0.034 (0.0397)	–	–
SARIMA models of *P. falciparum*											
Baseline (0,1,0) (1,1,0)_12_	–	−0.631 (0.1873)*	–	–	–	–		–	–	–	–
(1,0,0) + Tp [1,3,4] + Tmin [3] + Tmax [1,2,3]	0.744 (0.1543)*	–	–	0.001 (0.0007)	0.002 (0.0009)*	0.002 (0.0007)*		0.072 (0.0364)*	−0.046 (0.0450)	0.113 (0.0674)	−0.031 (0.073)

ar1 = autoregressive term; *P. falciparum* = *Plasmodium falciparum*; *P. vivax* = *Plasmodium vivax;* sar1 = the seasonal autoregressive term; SARIMA = seasonal autoregressive integrated moving average; sma1 = the seasonal moving average coefficient; Tmax[i] = the maximum temperature at lag I; Tmin[i] = the minimum temperature at lag i; Tp = total precipitation. The values inside brackets denote the standard errors of the estimated coefficients.

* Significance level = 0.05.

Initial cross-correlation exhibited strong correlations between malaria and precipitation, maximum temperature 0 to 4 months prior, and minimum temperature 1 to 3 months prior. However, after prewhitening, all cross-correlations disappeared, indicating that the apparent associations were driven by shared seasonal trends rather than independent lagged effects (Supplemental Figures 5–7).

Using the SARIMAX approach, implemented using the auto.arima function, all possible combinations of significant climate variables and time lags in the raw cross-correlation analysis were tested. For overall malaria, the best-fitting model included precipitation 1, 3, and 4 months prior, as well as maximum temperature from the same month and 2 months prior, with an autoregressive integrated moving average (ARIMA) (0,1,0) error structure. The effects of precipitation were consistently positive across lags, whereas the direction of temperature effects varied, with a negative association for maximum temperature in the same month and a positive association at lag 2. For *P. vivax*, the optimal model included precipitation 1 and 3 months prior and maximum temperature 2 months prior, along with an ARIMA (0,1,0) error structure. All retained covariates exhibited positive associations with incidence. For *P. falciparum*, the best model included precipitation 1, 3, and 4 months prior, minimum temperature 3 months prior, and maximum temperature 1, 2, and 3 months prior, along with an ARIMA (1,0,0) error structure. Precipitation was positively associated with incidence across lags, whereas the minimum temperature at lag 3 and maximum temperature at lag 2 were negatively associated; in contrast, the maximum temperature at lags 1 and 3 was positively associated. Overall, the results indicate that the effects of climate variables on malaria incidence were distributed across multiple lags, and the direction of association varied by both lag and parasite species.

Model performance improved notably with the inclusion of climate covariates: the out-of-sample MAE decreased from 0.35 to 0.25 for overall malaria, decreased from 0.31 to 0.21 for *P. vivax*, and decreased from 0.35 to 0.26 for *P. falciparum* (Table 3). The diagnostic checks of all models are presented in Supplemental Figures 8–10. The Ljung-Box tests yielded higher *P*-values (0.18 for overall malaria, 0.12 for *P. vivax*, and 0.18 for *P. falciparum*), indicating that the residuals approximate white noise and that the models adequately captured the temporal dependence in the data. The commune-level sensitivity analysis revealed consistent model performance, with scaled MAE values ranging from 0.065 to 0.096 for overall malaria, 0.123 to 0.184 for *P. vivax*, and 0.066 to 0.113 for *P. falciparum*. The direction and magnitude of the coefficients were also generally stable across communes (Supplemental Table 1).

**Table 3:** Out-of-sample performance comparison between models with and without covariates

Models	AIC	Ljung-Box Test	MAE	RMSE
Overall baseline	25.83	0.73	0.35	0.42
Overall with covariates	50.90	0.18	0.25	0.33
*P. vivax* baseline	4.27	0.56	0.31	0.43
*P. vivax* with covariates	20.63	0.12	0.21	0.27
*P. falciparum* baseline	22.16	0.07	0.35	0.41
*P falciparum* with covariates	32.28	0.18	0.26	0.32

AIC = Akaike information criterion; MAE = mean absolute error; *P. falciparum* = *Plasmodium falciparum*; *P. vivax* = *Plasmodium vivax*; RMSE = root mean square error.

**Figure 5.**
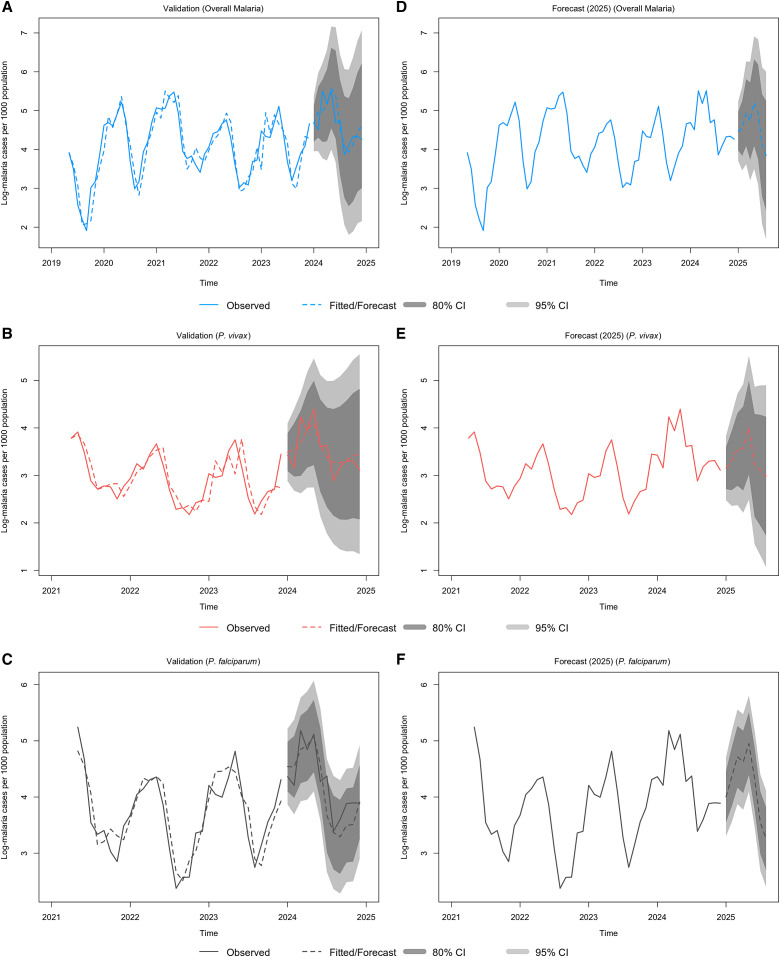
Fit of the separate seasonal autoregressive integrated moving average (SARIMA) model to malaria time series data. (**A–C**) Comparison between the observed data (solid line) and the predicted (fit, dashed line) values between January 2019 and December 2023, including 2024 data for validation, based on the best SARIMA model with covariates for (**A**) all malaria, (**B**) *Plasmodium vivax* (*P. vivax*), and (**C**) *Plasmodium falciparum* (*P. falciparum*). (**D–F**) Actual data for the study period (January 2019–December 2023) alongside the predicted values for the future period (January 2025–August 2025) and the corresponding 80% and 95% prediction intervals for (**D**) all malaria, (**E**) *P. vivax*, and (**F**) *P. falciparum*.

The predicted malaria incidences were relatively close to the observed values, indicating that models provided an acceptable fit for predicting malaria incidence within the study area. The forecasts for 2025 suggested a decline in malaria incidence for both overall malaria and individual species compared with 2024, although the predicted levels remained higher than those recorded in 2023. The models effectively captured the characteristic bimodal pattern of malaria incidence (Figure 5).

## DISCUSSION

Monthly malaria case data from 27 health centers across the Mandoto District (2019–2024) were analyzed along with meteorological data to understand transmission dynamics and forecast potential influences of changing climate conditions using descriptive, cross-correlation, and SARIMA forecast models. Malaria incidence exhibited substantial interannual variation over the study period rather than a consistent upward trend. The number of reported cases was lowest in 2019, followed by a marked increase between 2020 and 2021, after which incidence levels stabilized between 2022 and 2023, although they remained higher than in 2019. In 2024, incidence rose again to levels comparable to the 2021 peak. The western part of the district consistently had the highest case burden. The incidence of cases due to *P. vivax* and *P. falciparum* exhibited seasonal patterns, characterized by a 12-month bimodal cycle of annual fluctuations with peaks in January and May. These fluctuations were closely associated with precipitation and temperatures, with a higher incidence after periods of increased precipitation and warmer temperatures. The temporal distribution of cases reflects the biological sensitivity of malaria transmission to climate conditions that favor mosquito breeding and survival, as well as parasite development.

The number of reported malaria cases increased from 2019 to 2024, with the highest annual parasite incidence recorded in 2021. This peak in incidence may be attributable to the impact of the coronavirus disease 2019 (COVID-19) pandemic on healthcare systems. More people experiencing symptoms common to malaria and COVID-19 may have visited healthcare facilities to get tested, leading to a higher detection rate of malaria cases, as has been documented in previous studies.^45^^,^^46^ Additionally, the pandemic may have disrupted malaria control interventions, such as the distribution of insecticide-treated bed nets (ITN) and indoor residual spraying (IRS). During the COVID-19 pandemic, 40% of countries, including Madagascar, reported disruptions in the implementation of IRS and ITN distribution activities.^47^^,^^48^ The combination of increased healthcare visits and disrupted interventions likely created conditions favorable to the malaria spike seen that year. On the other hand, stockouts of RDTs were common across all health centers in Madagascar, which may have affected the accuracy of malaria case detection. However, detailed information on the frequency and duration of these stockouts is not available.

The seasonal patterns for both *P. vivax* and *P. falciparum* are consistent with a peak around January and February and a second, larger peak around April and June. This is consistent with the findings of the studies by Nguyen et al. and Howes et al.^21^^,^^49^ Applying a seasonal decomposition analysis to monthly malaria case proportions revealed that during the first half of the year, the distribution of cases due to *P. vivax* and *P. falciparum* is relatively balanced, whereas the second half of the year is predominantly driven by *P. vivax*. In the first peak during May, 14.2% of *P. vivax* cases and 15.2% of *P. falciparum* cases were observed. During the trough in August, 4.3% of *P. vivax* cases occurred, compared with 2.7% of *P. falciparum* cases. If both species had declined at the same rate after their peak, *P. vivax* cases would also be expected to reach comparably low levels in August. The greater proportion of *P. vivax* cases compared with *P. falciparum* cases during the dry season trough is consistent with the relapsing nature of *P. vivax*. Unlike *P. falciparum*, *P. vivax* can remain dormant in the liver as a hypnozoite and reactivate later, causing new infections without requiring recent exposure to infected mosquitoes. This biological characteristic allows *P. vivax* to persist even in seasons when mosquito populations are low.^50^^,^^51^ However, between August and November, mosquitoes were rarely collected, as noted in Tantely et al.^52^ Similar seasonal patterns have been observed in Ethiopia, where *P. vivax* relapses were identified as a significant contributor to case burdens outside of primary transmission seasons.^53^

Given the observed seasonal patterns in malaria incidence, targeted seasonal chemoprevention could be a valuable strategy for reducing the burden of malaria.^54^ In addition, improving diagnostic tools in areas where *P. falciparum* and *P. vivax* are co-endemic is crucial. The National Malaria Control Program plans to implement microscopy confirmation for RDT-positive cases to improve species identification and treatment accuracy. Accelerating the rollout of this microscopy component is strongly recommended because it would enhance the precision of species diagnosis and strengthen the quality of surveillance data. Given the differing pathologies of *P. vivax* and *P. falciparum* infections, recommended treatment strategies vary. *Plasmodium vivax* infections require additional drugs, such as primaquine, to target hypnozoites.^55^^,^^56^ Without species-specific diagnostics, the interactions between these two parasites remain obscured, hindering effective control strategies.

Incorporating climate variables significantly improved the accuracy of malaria incidence forecasting. Although correlation analysis initially revealed relationships between malaria incidence (both overall and species-specific) and precipitation and temperature, no statistically significant correlations remained after adjusting for seasonal and autoregressive patterns in the time series. This finding suggests that the climate plays a key role in driving seasonal dynamics in malaria transmission. This interpretation is supported by the SARIMA modeling results. In the baseline models without climate covariates, the best-fitting structures—SARIMA (0,1,0) (0,1,1)_12_ for overall malaria, SARIMA (1,1,0) (0,1,0)_12_ for *P. vivax*, and SARIMA (0,1,0) (1,1,0)_12_ for *P. falciparum*—required both seasonal and nonseasonal differencing, as well as seasonal moving average or autoregressive terms, indicating strong and regular seasonality in the incidence data. However, when climate covariates were included, specifically precipitation 1, 3, and 4 months prior and maximum temperature 0 and 2 months prior for overall malaria, the best models were simplified to nonseasonal ARIMA (0,1,0) for overall malaria and *P. vivax*, and ARIMA (1,0,0) for *P. falciparum*. The removal of seasonal terms suggests that these climate variables captured much of the intra-annual variation previously modeled by the seasonal components and provided a more realistic representation of seasonal malaria dynamics. Model performance improved accordingly: the out-of-sample MAE value decreased from 0.35 to 0.25 for overall malaria, decreased from 0.31 to 0.21 for *P. vivax*, and decreased from 0.35 to 0.26 for *P. falciparum*. These findings are consistent with those of previous studies across sub-Saharan Africa that have highlighted the strong influence of rainfall and temperature variability on malaria transmission, particularly in regions with seasonal or unstable transmission. In semi-arid and highland areas, rainfall often determines the timing and magnitude of mosquito breeding and transmission peaks, whereas temperature modulates parasite development rates within the vector.^57^ These improvements highlight the value of incorporating climate drivers into malaria forecasting frameworks, particularly in endemic regions where transmission is strongly seasonal. A similar relationship between rainfall lagged 1–4 months and malaria incidence have been observed in Ethiopia, Kenya, and southern Africa.^58^^–^^61^

The association between climate variables and malaria incidence was distributed across multiple time lags, and the direction of the effects varied by lag and parasite species. The results suggest that precipitation exerts a predominantly positive influence on malaria incidence, with the strongest effects observed at lags of 1 to 4. This pattern aligns with the expected biological and ecological mechanisms: increased rainfall creates breeding sites for *Anopheles* mosquitoes, thereby increasing vector density after a delay corresponding to mosquito development and parasite incubation periods. The temperature effects were more complex and varied across lags. Negative associations at lag 0 of maximum temperature may reflect the immediate adverse impact of excessively high temperatures on mosquito survival or parasite development. Conversely, positive associations at lag 2 suggest that moderate temperatures in the preceding months may have favored vector and parasite development, leading to increased transmission after a delay. This aligns with previous thermal performance studies, which indicated that malaria transmission is optimal between 16°C and 34°C, with 25°C being the ideal temperature for transmission.^13^^,^^62^ As global temperatures continue to rise, the authors of a WHO study have predicted an increased risk of vector-borne diseases such as malaria in Madagascar.^63^ Additionally, Ryan et al. predicted shifts in the geographic distribution of malaria, with some regions becoming more favorable for transmission because of higher minimum temperatures, whereas others may experience decreased transmission because of excessive heat, as estimated.^64^ According to that study, climate change is expected to shift seasonal malaria patterns in Madagascar from western to the eastern regions, with some districts that previously did not experience seasonal malaria beginning to report cases. According to the current study’s findings, although the western part of Mandoto remains the most affected, malaria has spread across all parts of the district. Differences between *P. vivax* and *P. falciparum* also emerged, with *P. vivax* exhibiting fewer lagged effects. This may be due to the ability of *P. vivax* to relapse from hypnozoites in the liver, which can reduce its dependence on recent climate conditions compared with *P. falciparum.*^65^^,^^66^ Given that multiple lag terms were included in the models and are likely correlated, these results should be interpreted as indicative of the direction and timing of associations rather than the magnitude of individual effects. The consistent model performance across communes further supports the robustness of the relationship identified between climate variables and malaria incidence at the district level.

Despite improvements achieved by including climate variables, first-order differencing (d = 1) remained necessary in models of overall and *P. vivax* incidence, indicating a nonstationary trend in the malaria incidence series. This suggests that climate factors alone could not fully explain long-term changes in the malaria burden. Other influences, such as variations in public health intervention coverage, healthcare access, reporting practices, or drug resistance, may contribute to underlying trends and fluctuations not captured by climate data.

A key limitation of the present study is that interventions implemented within the district during the study period were not considered because of a lack of detailed available data. This limits the authors’ ability to assess the impact of malaria control interventions and COVID-19.

Other limitations include the limited time series for which species-specific data are available, raising the possibility of overfitting. Data sparsity may also have led to substantial uncertainty in our predictions. Uncertainty intervals at both 95% confidence and 80% confidence are presented. Finally, the SARIMAX framework assumes a linear relationship between malaria incidence and climate covariates, whereas in reality, the effects of rainfall and temperature are often nonlinear and may include saturation effects.

## CONCLUSION

Malaria incidence increased over the 6-year study interval in Mandoto, a district in Madagascar’s central highlands, and seasonal patterns in incidence were driven by climate factors. Additionally, the co-endemic species *P. vivax* and *P. falciparum* exhibit varying patterns of transmission. This study reveals the utility of SARIMA models in understanding malaria dynamics. In conclusion, these findings suggest that monitoring cumulative precipitation and short-term temperature anomalies can provide valuable early warning indicators for malaria risk. Integrating these climate variables into routine surveillance could enhance the timing and targeting of vector control interventions, such as IRS. In addition, incorporating precipitation and temperature forecasts into the local malaria early warning system would support proactive resource allocation and improve preparation for potential outbreaks. These findings underscore the need for species-specific control strategies, particularly in high-risk western communes in Mandoto, as well as the importance of accounting for climate variability in malaria prediction and intervention planning. By focusing on these aspects, malaria control programs can more effectively reduce the burden of *P. vivax*, complementing efforts to control *P. falciparum* and ultimately contributing to a reduction in overall malaria incidence. Despite heterogeneity in the malaria burden between communes, no commune was classified as low risk during the study interval. Widespread malaria transmission throughout the region poses challenges for prioritizing resource allocation and implementing target interventions because all communities require intensive malaria control efforts.

## Supplemental Materials

10.4269/ajtmh.25-0329 Supplemental Materials
