# Supplementary material for: Association of Climate Variables with Plasmodium vivax and Plasmodium falciparum Malaria Cases in Mandoto, Madagascar: A Statistical Modeling Study
Source: Am J Trop Med Hyg. 2026 Feb 10;114(4):690–701. doi: 10.4269/ajtmh.25-0329 (PMC13045628; doi:10.4269/ajtmh.25-0329)
Supplement: Supplemental Materials [file tpmd250329.SD1.pdf]

Supplementary

Statistical analysis

Pre-whitening process

We applied a pre-whitening approach to identify which lags of the climate variables were most strongly associated with malaria incidence after adjusting for shared patterns of temporal dependence. In this approach, SARIMA models were first applied to each climate variable and then to the malaria incidence using the same model order. We analyzed the cross-correlation function between the residuals of these models to identify any lags where changes in the climate variable could explain unexplained variability in malaria incidence. Significant lags were then added as external predictors to the baseline SARIMA model. Before fitting the SARIMA model, the monthly incidence rate data was log-transformed. This log transformation helps stabilize the variance over time, making the data more suitable for modeling, as it reduces the impact of large fluctuations

SARIMA model

SARIMA's structure, is defined by parameters that capture non-seasonal and seasonal influences, and expressed as $(\mathrm{p,d,q})(\mathrm{P,D,Q})_s$

$$F(B)G(B^s)(1-B)^d(1-B^s)^D\ Y_t = H(B)K(B^s)\epsilon_t \quad (1)$$

Where B is the backshift operator, Y_t is the time series of malaria incidence data at time t, F is the Autoregressive (AR) polynomial of order p, G is the seasonal AR polynomial of order P, H is the Moving Average (MA) polynomial of order q, K is the seasonal MA polynomial of order Q, d is the degree of differencing, s is the seasonal period. In our case, $s = 12$ since we have monthly data. ϵ_t are error terms known as white noise. They are interpreted as an exogenous effect that the model is

not able to explain and may be contributions of other climates variables which are not included in the model.

Once the best SARIMA model was determined, we incorporated the climate variables at the identified lags, based on the pre-whitening process, to create a SARIMA with exogenous variables (SARIMAX). Using forecast package, SARIMAX is equivalent to a regression with ARIMA errors. Similarly, with the equation of SARIMA in (1), SARIMAX or regression with ARIMA errors model is simply defined as linear regression with ARIMA errors

$$Y_t = \omega X_t + \eta_t, \quad n_t \sim SARIMA(\mathrm{p,d,q})(\mathrm{P,D,Q})_s \quad (2)$$

$$F(B)G(B^s)(1-B)^d(1-B^s)^D\eta_t = H(B)K(B^s)\epsilon_t$$

where X_t represents a covariate at time t and ω is its coefficient. In regression with ARIMA errors model, the covariate coefficient has its usual interpretation. The inclusion of covariates in the SARIMAX (Regression with ARIMA errors) model allows us to assess whether the model's performance has improved, meaning, if the performances metrics (standard errors, AIC) indicate a better fit with the added variables.

Forecasting performance

The best model for forecasting was the model with the minimum mean absolute error (MAE).

$$MAE = \frac{1}{T}\sum_{t=1}^{T}|y_t - \hat{y}_t|$$

$$scaled\_MAE = \frac{MAE}{\max y_t - \min y_t}$$

Where y_t is the actual observation, $\hat{y}_t$is the fitted and T is the number of samples

Table S1 Model performance and meteorological covariate estimates for malaria incidence by species and commune

Metric	Anjoma Ramartina	Ankazomiriotra	Antanambao Ambary	Betsohana	Mandoto	Vasiana	Vinany
Overall – MAE-in-sample	0.3192	0.4826	0.4006	0.473	0.3924	0.5881	0.3556
Overall - Scaled MAE	0.096	0.076	0.086	0.094	0.085	0.092	0.065
Overall – Tp 1	0.0003	0.001	0.0015*	0.0009	0.0013	0.0018	0.0015*
Overall – Tp 3	0.0007	0.0014	0.0009	0.0018*	0.0007	0.0004	0.0016*
Overall – Tp 4	0.0013*	0.0026*	0.0022*	0.0015	0.0018*	0.0025*	0.0028*
Overall - Tmax0	-0.0108	-0.0031	-0.1144*	-0.0352	-0.068	-0.0365	0.0444
Overall - Tmax2	0.1261*	0.2150*	0.032	0.1263	0.1063*	0.1671	0.1083*
P. vivax - MAE-in-sample	0.2503	0.6398	0.4249	0.5557	0.4327	0.4344	0.464
P. vivax - Scaled MAE	0.158	0.123	0.181	0.146	0.147	0.184	0.172
P. vivax – Tp 1	-0.0002	0.0003	-0.0001	-0.0024	0.0014	0.0005	0.0025
P. vivax – Tp 3	0.0011*	0.0022	0.0013	0.0018	0.0008	0.0006	-0.0008
P. vivax - Tmax2	0.0098	0.0188	0.0443	0.0377	0.0451	0.0747	0.0456
P. falciparum - MAE-in-sample	0.2954	0.3915	0.2886	0.3639	0.3217	0.2846	0.3148
P. falciparum - Scaled MAE	0.113	0.095	0.093	0.102	0.11	0.084	0.066
P. falciparum - AR1	0.4848*	0.4833*	0.6145*	0.4712*	0.3739	0.6005*	0.9182*
P. falciparum – Tp 1	0.0007	0.0013	0.0015	0.0001	-0.0001	0.0015	0.0021*
P. falciparum – Tp 3	0.001	0.0012	0.0003	0.0040*	0.0012	0.0012	0.0017
P. falciparum – Tp 4	0.0014	0.0022	0.0023*	0.001	0.0006	0.0025*	0.0023*
P. falciparum - Tmax1	0.1691*	0.1077	0.1632*	0.0877	0.0363	0.0136	0.0816
P. falciparum - Tmax2	-0.0766	0.0527	-0.0538	-0.0656	0.0409	0.0344	-0.0161
P. falciparum - Tmax3	0.0779	-0.0139	-0.0227	0.1543	0.0434	0.2127*	0.0306
P. falciparum - Tmin3	0.0373	0.0954	0.1792	-0.0439	0.0877	-0.0383	0.0207

Results shown for overall malaria (ARIMA (0,1,0)), *P. vivax* (ARIMA (0,1,0)), and *P. falciparum* (ARIMA(1,0,0)) models. MAE = Mean Absolute Error. Significance levels: * $p < 0.05$, Overall model uses complete dataset; species-specific models use January 2021 - October 2023. Tp means total precipitation. Fidirana has been excluded from the analysis due to insufficient data.

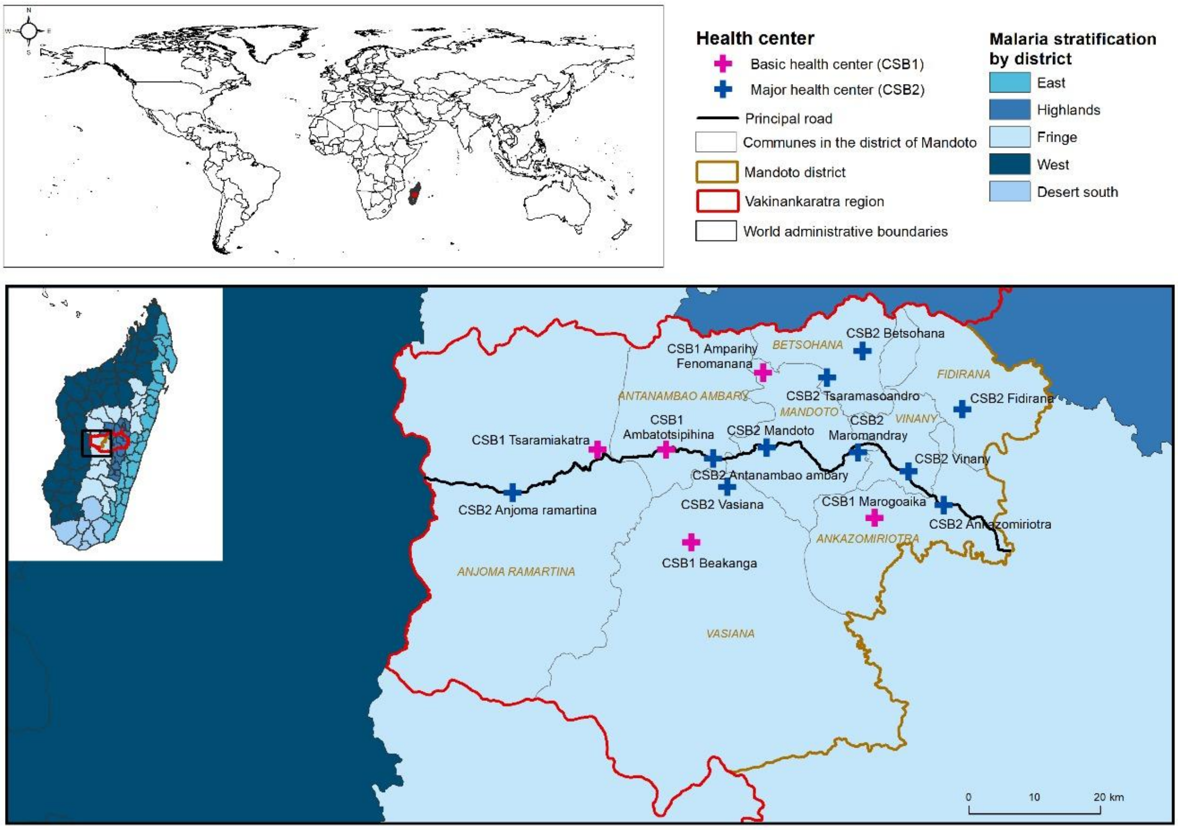

Figure S1. Mapping of the eight communes in Mandoto district of Madagascar with major (blue, CSB2) and basic (pink, CSB1) health center locations labelled. CSB refers to a primary health center (Centre de Santé de base in French). The inset at the top left shows the global location of Madagascar, while the second inset highlights Madagascar with the Vakinankaratra region outlined in red.

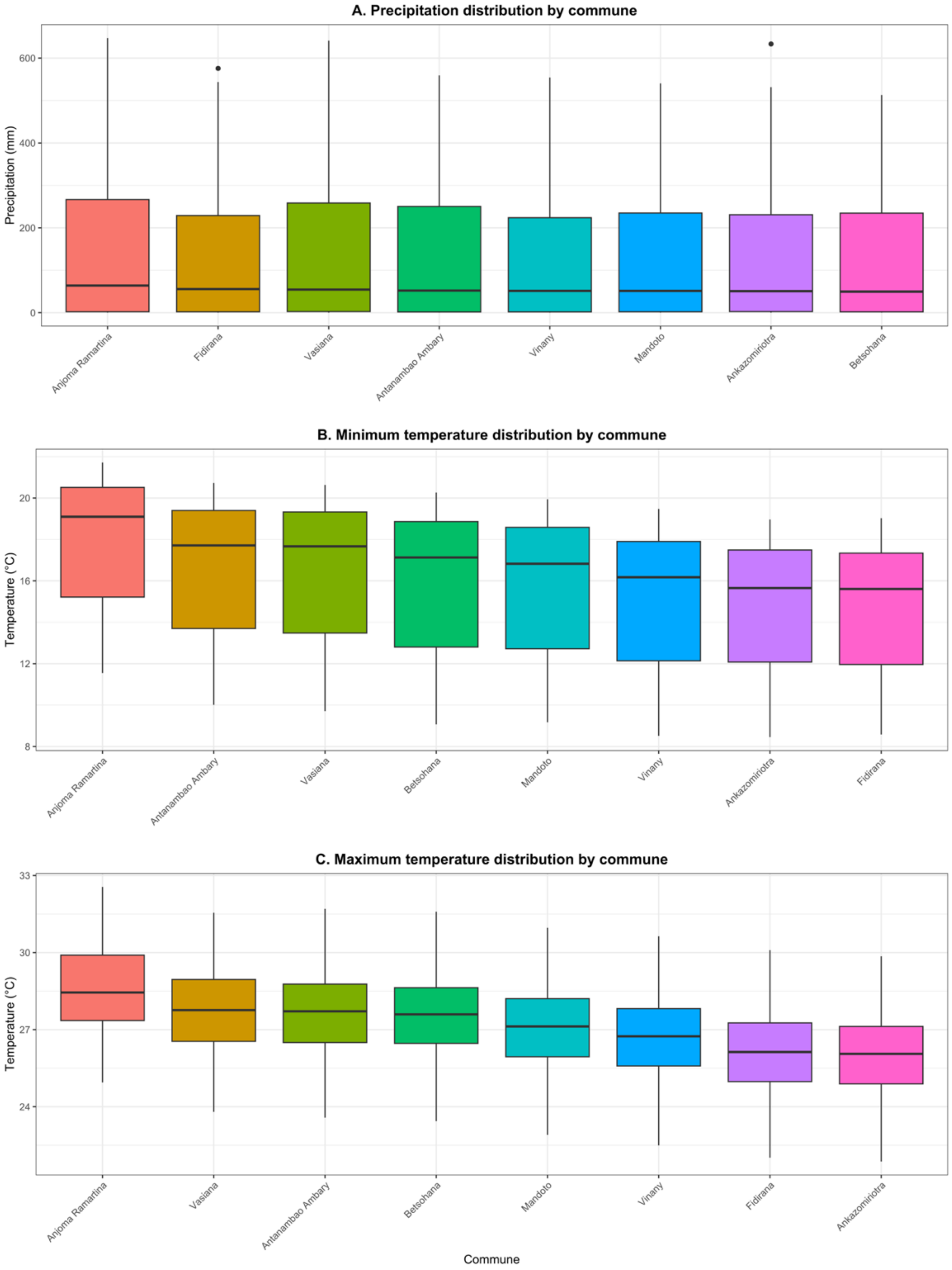

Figure S2. Box plot of precipitation and temperature by commune in Mandoto district. The western communes show higher median of maximum and minimum temperatures compared to eastern communes, indicating a clear east-west temperature gradient.

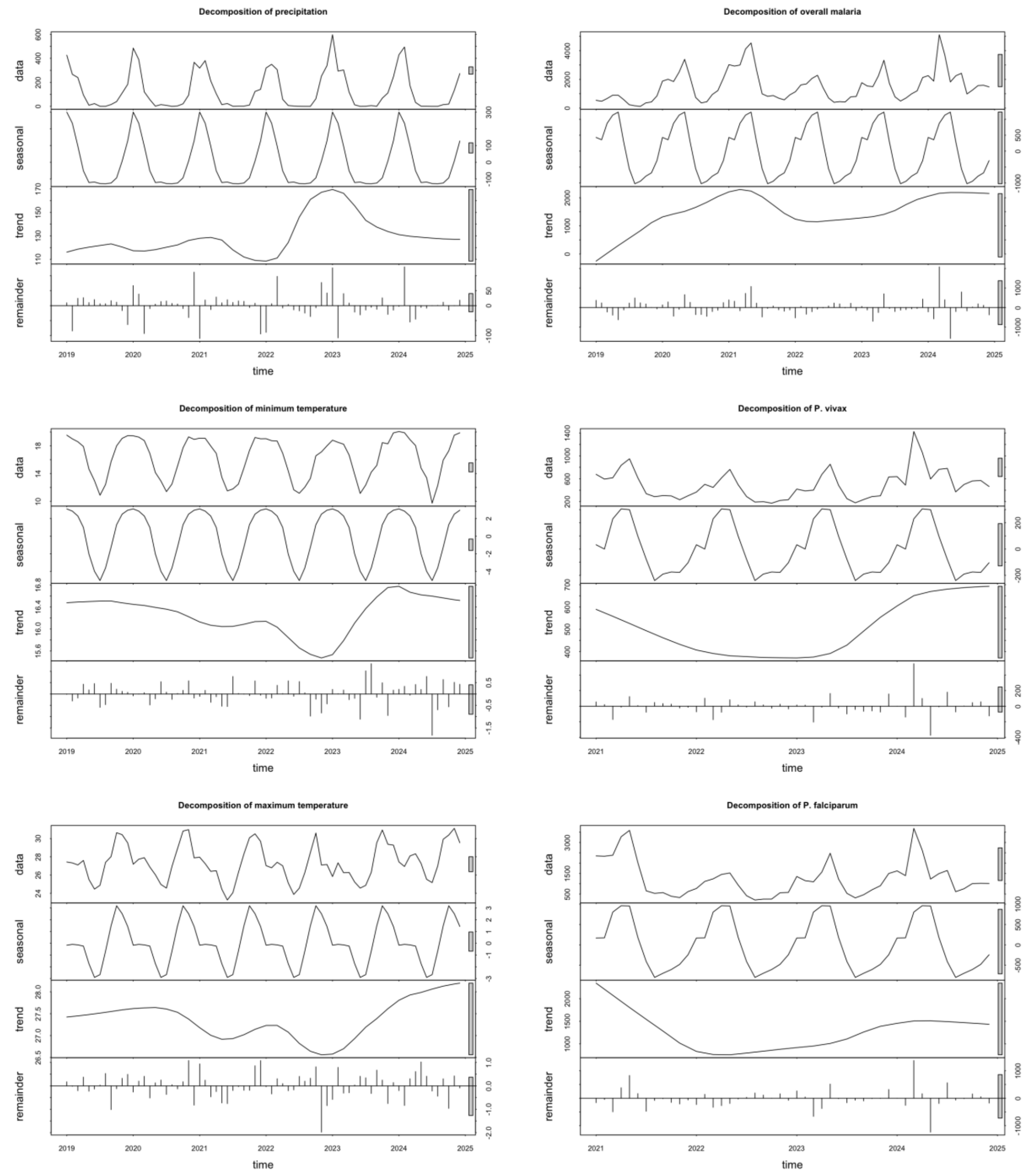

Figure S3. Decomposition of all the time series data. This illustrates the Seasonal-Trend Decomposition based on Loess (STL) applied to the time series data. Each panel shows the original time series, along with its decomposed components: the long-term trend (capturing the underlying direction over time), the seasonal component (representing the recurrent patterns within the year), and the remainder (unexplained variation or irregular noise).

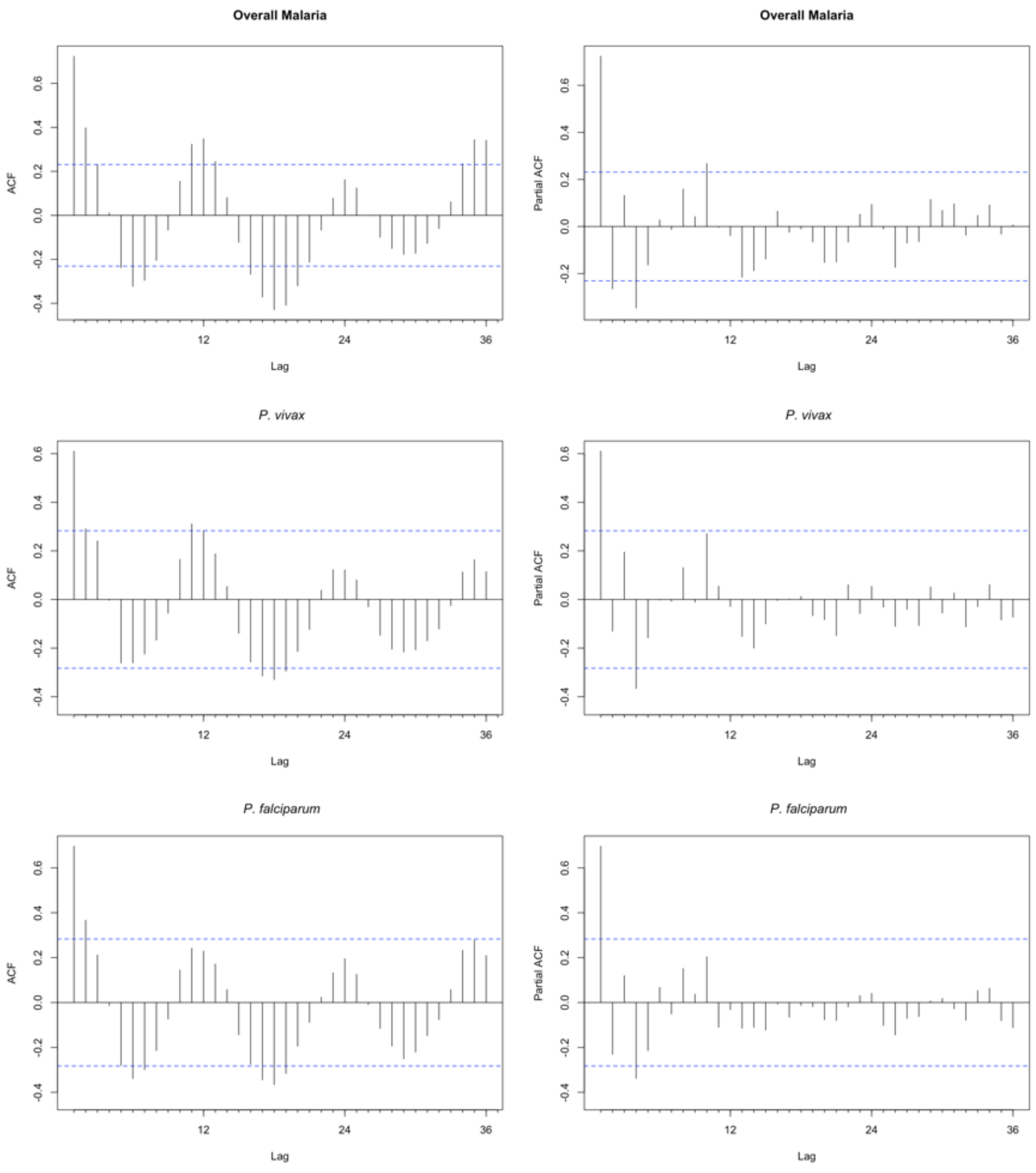

Figure S4. ACF and PACF plot of monthly malaria case counts. The first line represents the overall malaria case count from January 2019 to December 2024. The second and third lines show *P. vivax* and *P. falciparum* case counts separately from January 2021 to December 2024.

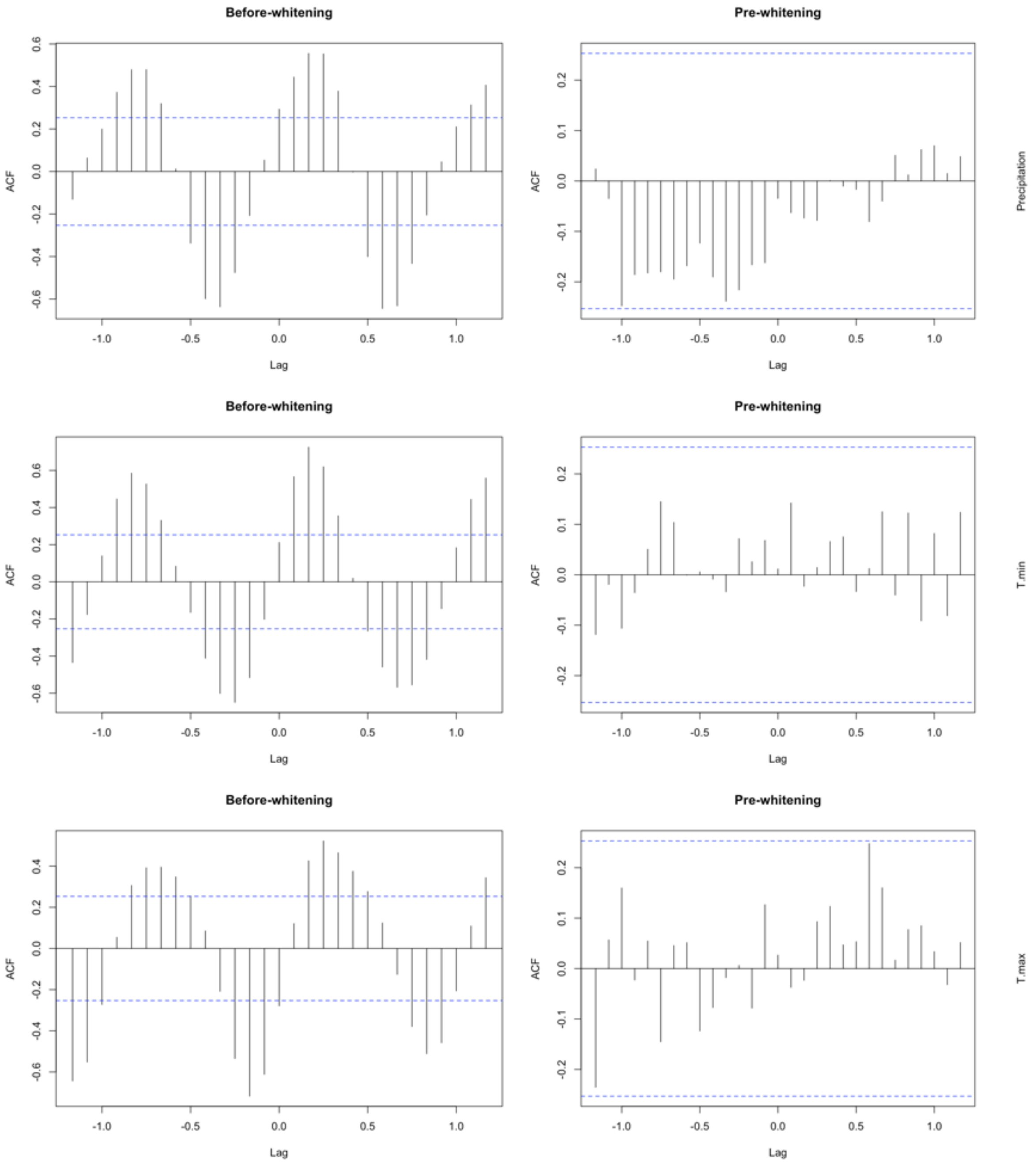

Figure S5. Cross-correlations between overall malaria incidence and meteorological variables. The left panels show Cross-correlation results before whitening, while the right panels display cross-correlation after the whitening process, which adjusts for autocorrelation in the time series.

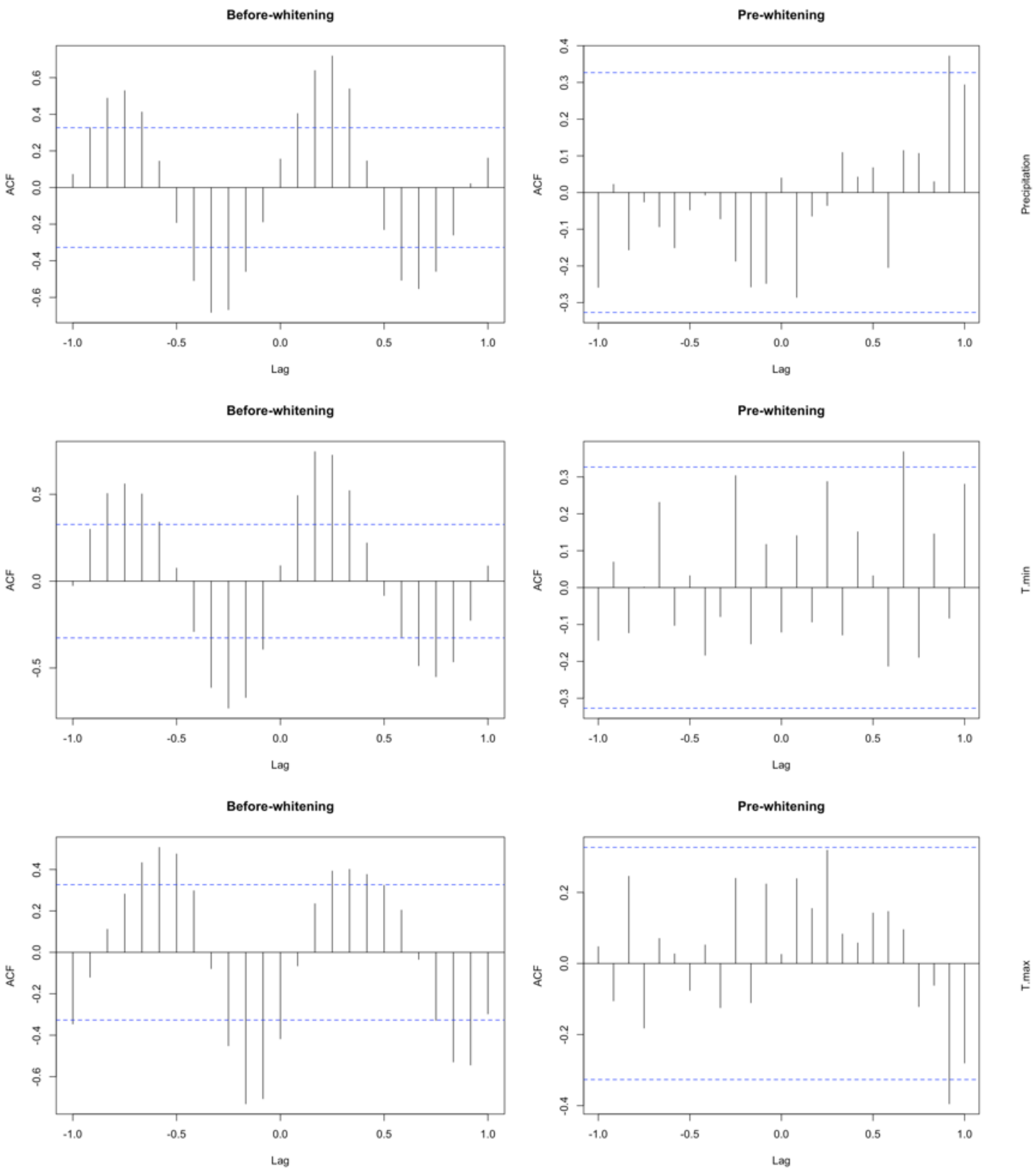

Figure S6. Cross-correlations between *P. vivax* malaria incidence and meteorological variables. The left panels show Cross-correlation results before whitening, while the right panels display cross-correlation after the whitening process, which adjusts for autocorrelation in the time series.

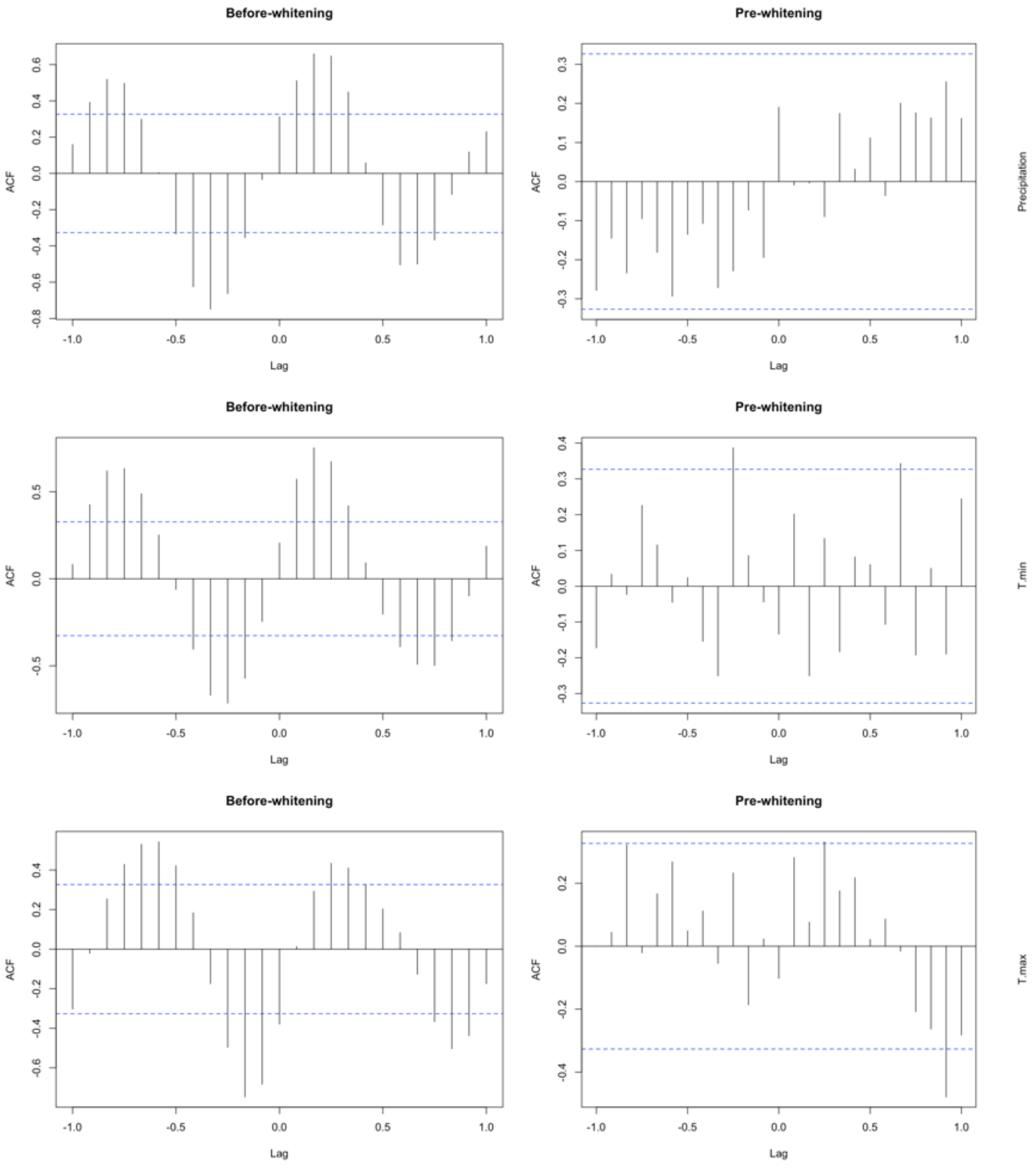

Figure S7. Cross-correlations between *P. falciparum* malaria incidence and meteorological variables. The left panels show Cross-correlation results before whitening, while the right panels display cross-correlation after the whitening process, which adjusts for autocorrelation in the time series.

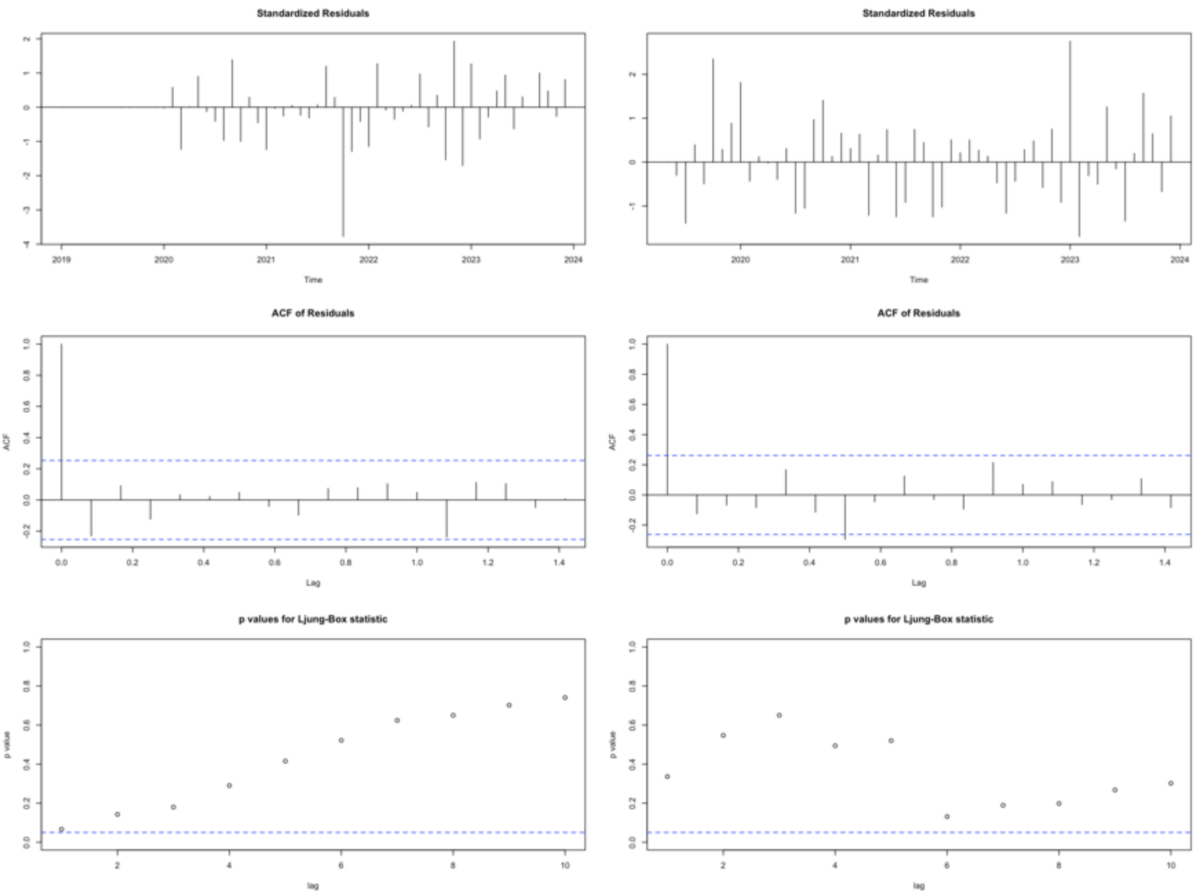

Figure S8. Residuals plots of models fitted to the transformed overall malaria incidence. The left panel show residuals from the model without covariates, while the right panel shows residuals from the model with covariates.

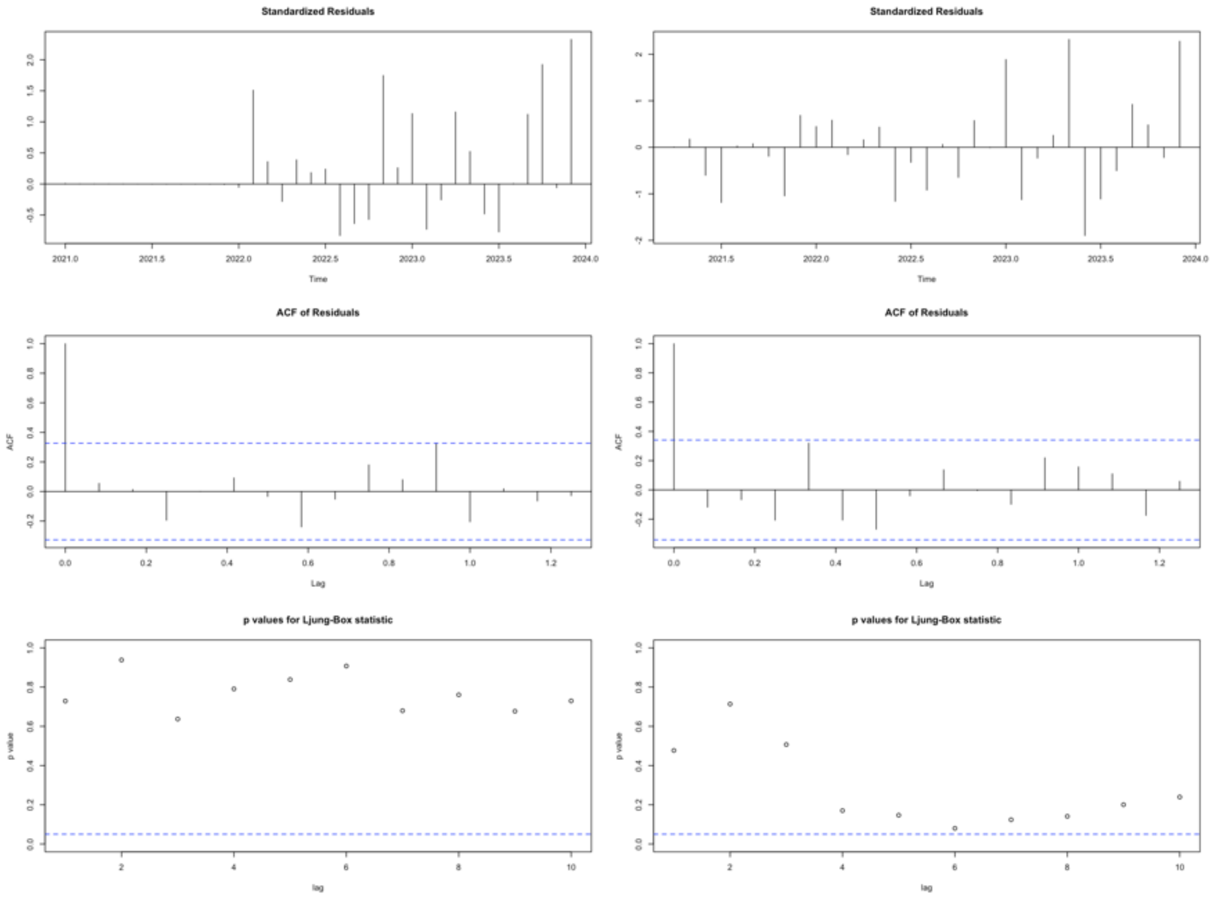

Figure S9. Residuals plots of models fitted to the transformed *P. vivax* malaria incidence. The left panel show residuals from the model without covariates, while the right panel shows residuals from the model with covariates.

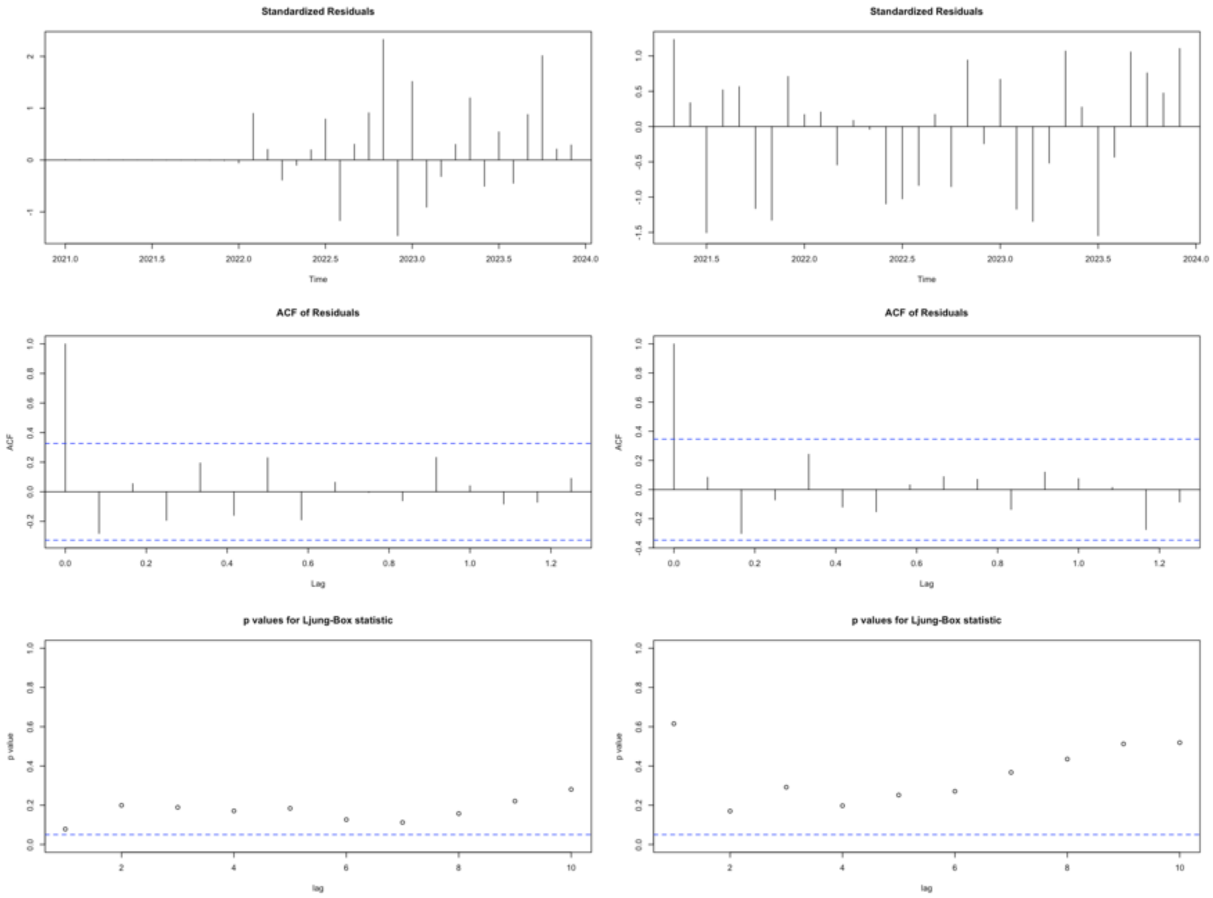

Figure S10. Residuals plots of models fitted to the transformed *P. falciparum* malaria incidence. The left panel show residuals from the model without covariates, while the right panel shows residuals from the model with covariates.
